# Understanding the impact of unmet needs on the health and wellbeing of people with stroke, living in the community: a scoping review of reviews

**DOI:** 10.3389/fneur.2026.1861343

**Published:** 2026-10-13

**Authors:** Susan Hillier, Carol McKinstry, Dominique A. Cadilhac, Hanan Khalil, Anna Joy, Leeanne M. Carey

**Affiliations:** 1 IIMPACT, Adelaide University, Adelaide, SA, Australia; 2 Rural Health School, La Trobe University, Bendigo, VIC, Australia; 3 Violet Vines Marshman Centre for Rural Health Research, Bundoora, VIC, Australia; 4 Department of Medicine, School of Clinical Sciences at Monash Health, Monash University, Clayton, VIC, Australia; 5 Stroke Theme, The Florey Institute of Neuroscience and Mental Health, The University of Melbourne, Heidelberg, VIC, Australia; 6 Department of Public Heath, School of Psychology and Public Health, and Care Economy Research Institute (CERI), La Trobe University, Melbourne, VIC, Australia; 7 Occupational Therapy, School of Allied Health Human Services and Sport, La Trobe University, Bundoora, VIC, Australia; 8 Occupational Therapy, School Primary and Allied Health Care, Monash University, Frankston, VIC, Australia; 9 Occupational Therapy, School of Allied Health Human Services and Sport and Care Economy Research Institute (CERI), La Trobe University, Bundoora, VIC, Australia; 10 Neurorehabilitation and Recovery, The Florey, The University of Melbourne, Heidelberg, VIC, Australia

**Keywords:** health, scoping review, stroke, unmet needs, wellbeing

## Abstract

**Background:**

Recovery from stroke may often be incomplete, with unresolved issues persisting as unmet needs. However, the impact of these needs on both the health and wellbeing of people with stroke, subgroups, and their carers remains unclear.

**Objectives:**

This scoping review of reviews aimed to provide an overview of evidence on unmet needs and their impact on the wellbeing of people living in the community with stroke.

**Methods:**

Relevant databases (14) were searched from 2,000 onwards and included systematic, scoping, and umbrella reviews meeting criteria related to population, concept, and context, and reporting quantitative and/or qualitative data.

**Results:**

Over 620 studies (including repeated studies across reviews) and 3.5 million participants were described in 28 eligible reviews. The needs and impacts were wide-ranging for the population as a whole and for sub-groups, e.g., with specific impairments (communication issues) or demographics (younger people who may have had a stroke or people who are culturally and linguistically diverse). No one framework covers the diversity of impacts on health and wellbeing—physical, emotional, and psychological health, as well as issues of relationships, inclusion, engagement, meaning, cultural and spiritual needs, financial, and other environmental domains.

**Conclusion:**

Our findings provide important, often ignored insights and direction for people with stroke generally, as well as sub-groups. Ultimately, services and research should operate at multiple levels and address quality-of-life impacts, including social integration, sense of self, and life purpose.

**Systematic review registration:**

Review protocol registration: https://osf.io/efut8/.

## Introduction

Rapid advances in hyperacute medical care have led to improvements in survival and disability outcomes for people after stroke. It is recognized that with shortening lengths of stay (e.g., average 4 days in the United States after acute stroke (1) or median 5 days in Australia (2)), recovery and community re-integration remain incomplete for many as they return home, often with disability, from the healthcare system. Care transitions, which occur when care setting change, represent an especially vulnerable period for survivors (3). For example, after stroke, returning home may lead to various challenges, including practical, psychosocial, and health-related unmet needs that may also impact caregivers (3, 4). Underserved and minority populations are especially vulnerable to the problems that may arise after returning to the community, as a consequence of poor environmental, social, and economic conditions, or different forms of racism (e.g., structural discrimination) (3). The ability to adjust after returning home following a stroke is multifaceted and is influenced by beliefs about capabilities in performing everyday activities, and levels of resilience and self-efficacy (5). Unmet needs, emerging from unresolved issues, are very prevalent across different domains because of the diversity of factors impacting people who survive a stroke.

Unmet needs include needs for healthcare services, as well as needs for social care and other supports (6). The perception of what needs are ‘unmet’ has been reported to differ across different stakeholders, including the person with stroke, healthcare professionals, or caregivers. Unmet needs have been well documented across several systematic reviews of various stroke populations. The recent review by Chen et al. (6) reported that the median number of unmet needs per stroke survivor was between two and five, with 73.8% having at least one. These authors found the largest number of unmet needs perceived by stroke survivors related to environmental needs (i.e., support, services, safety, accessibility/accommodation), followed by body functioning (i.e., psychosocial function, physical function, cognitive function) and then activities/participatory needs (i.e., healthy lifestyle, physical activities, independence). There were also commonalities in unmet service needs such as information needs, transport, home help/personal care, and therapy. However, it remains unclear what the impact of these unmet needs is on the health and wellbeing of the people concerned, the significance of the impact, and in what domains.

We undertook a scoping review of reviews to map evidence describing the unmet needs experienced by people after stroke, and how these impact their health and wellbeing in the community. We used a scoping review methodology to clarify key concepts in the literature, identify features of the concept of unmet needs and impact, and to identify knowledge or practice gaps (7). This information will inform solutions for adopting a more positive focus on wellbeing, while also being tailored to address unmet needs based on different stroke survivor profiles or other factors.

### The overall objectives of this scoping review include

1. To provide an overview of documented unmet needs for people after stroke.
2. To identify the impact of these unmet needs on the health and wellbeing of people with stroke living in the community, and describe any barriers and facilitators to these impacts.
3. To identify sub-groups who may experience different unmet needs/impact based on their profile or other factors.

## Methods

### Study design

We conducted a comprehensive scoping review of reviews. Our methods and reporting conform to the PRISMA extension for Scoping Reviews (8), and the methodology published by Peters et al. (9) The protocol (for populations of stroke, traumatic brain injury, and cancer-related brain damage) was published in the Open Science Framework (September 2022 https://osf.io/efut8/)—this paper reports on the stroke-only section of this protocol.

### Eligibility criteria for the included studies

#### Population

Included: Adults with subacute or chronic stroke; studies with multiple stakeholders (e.g., carers, health professionals, and people living with acquired brain injury) were only included if the data could be extracted separately for those living with a stroke, their caregivers, and health professionals.

Excluded: Studies with participants younger than 18, at the acute stage of their stroke (within 4 weeks of event/diagnosis), and/or with progressive or other neurological conditions (e.g., Parkinson’s disease, dementia).

#### Concept

Included: Both unmet needs and the impact on the health and wellbeing of people with stroke living at home and in the community, in association with:

- Latency (from sub-acute through to chronic stages, or at transition stages)
- Subgroups of impairments (e.g., communication, sensory, cognitive, and/or physical)
- Geographical or cultural sub-groups
- Prediction models or described associations with unmet needs and outcomes that reflect impacts on health and wellbeing.

Excluded: Insufficient detail to inform our understanding of the concepts of unmet needs and impact on health and wellbeing.

#### Context

Included: Community setting—own homes, in the community; data with participants reflecting on their community experience at any time after the acute phase has passed.

Excluded: Living in residential care or in hospital/inpatient care at the time of data collection.

### Types of evidence source

Systematic or scoping reviews were included to capture the broadest extent of unmet needs and their impact. We considered both quantitative data (such as the number and type of unmet needs and their impact on quality of life or participation and/or activity scores) and qualitative data (self-reports of impact on health and wellbeing) but reported both descriptively. We included only reviews (systematic or scoping) given the high number of potential inclusions.

We were restricted to the English language only, with a publication date range of 1 January 2000–30 February 2026, to investigate *contemporary* understandings of unmet needs.

### Search strategy

We searched databases (Medline, EMBASE, Health-Society, Humanities & Social Sciences Collections, PsycINFO, ProQuest (Social Science), Cochrane, SCOPUS, CINAHL, Academic Search Premier, Health Source (Nursing/Academic Edition), Psychology and Behavioural Sciences Collection, and SAGE (Health Sciences)) in October 2022, July 2024, and February 2026. We also searched the reference lists of the included studies.

The search strategy was developed for the MEDLINE database and was adapted for other databases. An example of a search strategy constructed with input from an academic librarian is in Appendix 1. Full search terms are available from the authors.

### Screening the studies

We imported search results directly from the databases into Covidence, where duplicates were automatically removed. Two authors reviewed/screened the titles and abstracts, and then the full texts. Conflicts were resolved by a third author. We used the Covidence flowchart to track the inclusion/exclusions of studies.

### Data extraction

A customized Excel spreadsheet was developed for data extraction, with two people extracting and cross-checking. The information extracted included:

- Citation details: author/s, date, title, journal, volume, issue, pages
- Country
- Population:
  ◦ Participants: age/sex and number
  ◦ Type/classification of stroke
  ◦ Time since injury: sub-acute v chronic; transition phase/point
  ◦ Whose perspective: person/carers/family/health provider
  ◦ Geographic or cultural sub-group
  ◦ Impairment sub-group: cognitive, communication, physical, etc.
- Concept
  ◦ Unmet need/s: needs for healthcare services, social supports, etc. (6)
  ◦ Impact: the domains of health and wellbeing that are impacted by different types of unmet needs and overall unmet needs
  ◦ Facilitators and barriers to health and wellbeing
  ◦ Application of predictive modeling and multimodal approaches to predict health and wellbeing.
- Context – of the unmet need/s and the impact according to:
  ◦ The person with the acquired brain injury
  ◦ The carers and supporters
  ◦ Providers (e.g., health professional, organization)
  ◦ The health and community ecosystem
  ◦ Year of data collection and whether cross-sectional or repeated measures in the same study cohort.

### Quality/risk of bias appraisal

We did not conduct a risk of bias or quality appraisal because this scoping review of reviews aimed to establish the breadth of evidence (and gaps) and identify areas for further research, rather than focus on the quality of existing evidence (8).

### Data synthesis

A deductive approach was used to map the results of the current review using the WHO International Classification of Functioning (ICF) (10) and wellbeing indicators (11). Extracted data from the included articles were themed and mapped into a broad biopsychosocial framework (12) to determine the impact on biological, psychological, or social domains. Then the extracted unmet needs were mapped to the ICF domains as related directly to the pathology level (stroke), personal or environmental factors, and impacts on body structure/function, activity, or participation levels. Then we applied the definitions of wellbeing to map the presence of each indicator in each review. Mapping of the results onto these well-characterized frameworks was conducted by a research assistant and the first author for consistency, and checked by a second author. Excerpts from the reviews were extracted verbatim and highlighted the relevant words. These were deductively assigned individually to the cogent domains or indicators in each framework. Consistently repeated terms were kept as individual assignments to indicate their relative prevalence in the domains. The authors’ team discussed discrepancies. We differentiated between unmet needs and impact based on the descriptors used by the original authors.

A further analysis of the reviews was conducted (not specified in the registered protocol) to determine the degree of overlap among the included studies. The corrected covered area (CCA) was calculated as per Pieper et al. (13)

## Results

The searches yielded 28 papers that met the inclusion criteria for our scoping review (3,754 identified from databases; 964 duplicates removed, leaving 2,790 to be screened by title and abstract, of which 2,731 were excluded; 59 were screened by full text, with 31 excluded) (Figure 1). These comprised 3 umbrella reviews, 14 systematic reviews, seven scoping reviews, with the remainder including mixed, qualitative, or rapid evidence reviews. The number of studies and included study designs varied markedly. Some authors did not report the study type, but those that did used qualitative, quantitative, and mixed-methods designs. Study designs included randomized controlled trials, prospective studies, cross-sectional studies, case–control studies, case reports, abstracts, and a letter to the editor. The number of studies included in the reviews ranged from 7 to 106. The dates of publication ranged from 2009 to 2024, with the majority (14) published between 2021 and 2024. Together, the reviews included summary information from over 620 studies (with duplicates across the reviews) with more than 3.5 million participants. However, the authors of 12 reviews did not report the total number of participants in their included studies.

**Figure 1:**
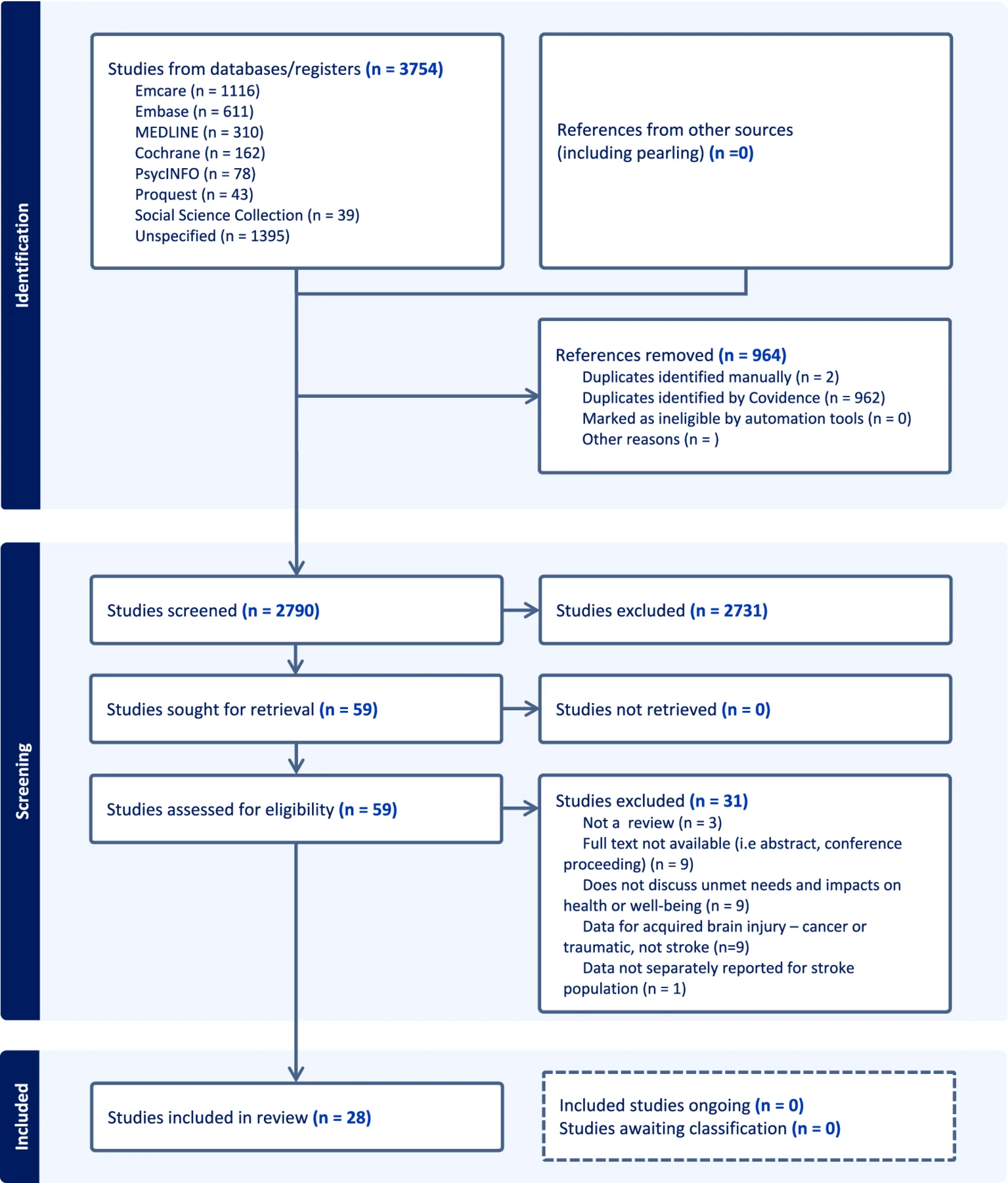
PRISMA flowchart.

### Study characteristics

Table 1 summarizes the 28 included reviews. Most studies came from Western countries (the United Kingdom, the United States, and Europe), with further representation from Asian countries, Australia/New Zealand, the Middle East, and West Africa.

**Table 1:** Summary of included reviews: review characteristics; extracted data (verbatim) related to unmet needs, impacted health, and wellbeing domains; identified facilitators and barriers to wellbeing and any relevant overall conclusions.

Author date [ref]	Review *N* studies	Population (subgroup) Perspective (*N*)	Unmet needs identified (i.e., physical, social, emotional)	Health and wellbeing domains impacted by unmet needs	Facilitators or Barriers	Overall conclusion
Assefa 2023 (32)	ScopR 13	Stroke (in Africa) PLWS (n/r)	Inadequate and/or inaccessible environment	Participation—as affected by environmental factors	Facilitators: immediate family and HPs; Barriers: products and technology, natural environment and human-made changes, attitudes, service systems and policies	Need to address barriers
Bright 2023 (33)	Qual SR	Stroke (living in NZ) PLWS (249)	Psychological	Mood, social relationships, self-identity, engagement in meaningful activities	Facilitator: psychosocial support Barrier: Lack of connection with family or with cultural groups	Wellbeing is individual but also collective. Connections with self, others, community and culture impact on wellbeing and influence support provided by stroke services
Chen 2019 (6)	SR 19	Stroke PLWS (37,383)	Unmet body functioning needs—20 sub-categories; Unmet activity/ participatory needs—22 subcategories; Unmet environmental needs—32 sub categories (detail provided on pages 4–7; Table 2)	All domains	None stated	Unknown association between assessment of unmet needs and provision of cost-effective interventions.
Choo 2023 (15)	UmbSR 10	Stroke (Chronic) Carer (2,552)	Disruption to psychological homeostasis; transitional uncertainty and loss; emotional burden and disguising vulnerability; obligatory caregiving, ill-preparation and inaccessible resources	Psycho-social- physical domains, e.g., Disruption to roles, diminishing self-identity, disruption to relationships, disempowerment, marginalization	Facilitators: coping mechanisms, recalibrate normality, balance, peer alliances, respite, mindful living, meaning-making, identifying growth, holistic support	Can be a painful and lonely journey—multi-faceted challenges. Need to process & re-frame lived experience—establish mutuality, compassionate alliance
Clark 2022 (20)	SR 20	Stroke (Limited English) PLWS (3million+)	Equitable care is lacking because of a lack of interpreters across services	Across domains	Barrier: lack of interpreters	
Connor 2023 (34)	SR	Stroke PLWS, caregivers, HPs (658)	Lack of knowledge about stroke, causes, secondary prevention, and medication. Generic information not sufficient. Inadequate data sharing, lack of carer support	Emotional, psychological, cognitive. Social and vocational activities received insufficient attention	Facilitator: good communication (interpersonal and organizational); HP knowledge and networks Barriers: how to access information and support	Transition times particularly problematic
Coutts 2023 (35)	ScopR 106	Stroke (RTW) PLWS (n/r)	Ability to return to work	Personal, social and vocational	Facilitators: socio-economic factors (age, gender, education, impairments) Barriers: work stress/demands, lack of awareness and resources, not adaptable.	Lack of research on interventions to support RTW. Lack of research on the impact of communication disorders on RTW.
Denham 2022 (36)	SR 13	Stroke Informal carers (n/r)	Inadequate information for carer education and training, lack of support in home care and services; how to take care of oneself and balance self with PLWS, financial support, service access	Emotional and psychological – coping and managing; relationships changing, social support	Facilitators: access	Complex and long-term needs
Greenwood 2009 (37)	SR 17	Stroke carers (272)	Carers’ training and own emotional responses	General	None stated	Carers wellbeing is important—positive and negative experiences
Guo 2021 (14)	SR 24	Stroke PLWS and carers (28)	Disease-related information—prognosis; prevention; treatment; symptoms. Information on communication with professionals and peers. Unmet activity/participation needs. Unmet physical recovery needs. Unmet family, community and social needs for environmental resources. Unmet rehabilitation plan, education and intervention needs. Unmet individual psycho-emotional support needs	Confidence, isolation; impact on physical and psychological recovery and impairment. Unmet social participation needs may result in embarrassment, shame, stigma, lack of connection/involvement in activities	Barrier: participants living with incorrect or limited information; ongoing feelings of uncertainty and distress about the management of symptoms following discharge.	Highlights the need to build a practical connection to meet the authentic needs of stroke survivors.
Halabi 2022 (38)	LR 37	Stroke PLWS (n/r)	Functioning needs; activities/participatory needs; environmental needs; service needs; home help/personal care; Information needs; Transport needs; therapy needs; emotional problems Fatigue. Poor health services/health needs (i.e., pain, dependency, depression)	Symptom burden (health), QoL	Facilitator: Knowledge and information, care coordination, community resources—palliative care services integrated with conventional care Barrier: Lack of support and resources, stigma, faith, and culture	Different unmet needs for young people < 45 years relating to QoL, loss of mobility, impact on social functioning, and marital conflicts.
Kokorelias 2023 (39)	ScopR n/r	Stroke PLWS, carers, family, HP (n/r)	Information needs, awareness of health service provider roles, accessing appropriate support	Exercise and diet (health)	Facilitators: community support and lifestyle, continuity of care, adequate information for adaptable changes Barriers: community stigma, language barriers, uncertainty	Need for culturally appropriate stroke services across the health care continuum for South Asian communities; educate HPs
Kristo 2022 (40)	RER 29	Stroke (aphasia) PLWS (n/r)	Mental health: sense of self; depression; anxiety; negative mood changes; trauma; loneliness.	Mental health: leads to long-term disability; affects wellbeing; increased risk of stroke	Facilitator: Involvement of social workers on the MDT as well as SLP and OT	Increase mood screening and psychological care for aphasic survivors to identify and reduce mental health issues
Lafiatoglou 2021 (41)	SR 17	ABI and stroke PLWBI (211)	Autonomy, decision-making; control post-acute phase	Dissatisfaction; disillusionment; anxiety; disempowerment; inhibit their potential to self-regulate and self-monitor their physical activities; sense of wellbeing;	Facilitators: efficient, effective communication, emotional support, family involvement in the rehabilitation process, creating a stimulating, enriching social environment/ease; humanize; facilitate older individuals’ adjustment to new reality. Barrier: illusionary unfulfilled return to socially constructed normality; another layer of difficulty for older individuals, negatively affecting their sense of wellbeing.	None relevant
Laurie 2023 (42)	ScopR 52	Stroke/TBI (experiences after discharge from inpatient rehab) PLWBI and Carers (4,137)	Lack of specialized knowledge from HPs; need for trusting relationships; inability to adapt; hard to navigate; not integrated and insufficient	Nothing specific	Facilitators and Barriers: characteristics of services: provider expertise, interpersonal qualities, partnership and adaptability; Characteristics of the health system: navigable system, integrated care, adequacy, and opportunity.	Need greater emphasis on personalized access to post-acute services—multitude of factors
Manning 2019 (18)	SR 31	Stroke (aphasia) PLWS (499)	Need for formal emotional support; Need for life-relevant therapy; Need for information about aphasia and treatment to better understand and explain their condition to others, to organize and access services and to be actively involved in decisions about their care	Loss of friendships; social isolation; negative impacts on relationships; lack of acceptance; loss of reciprocity; roles (i.e., parenting); loss of employment; sense of self and identity; financial impacts; social participation; uncertainty; ashamed; embarrassed.	Facilitator: proactive about participation; socializing; open to doing things differently; providing opportunities to develop confidence, autonomy, and skills; re-evaluating sense of self; progressing to higher levels of activity. Increased awareness of aphasic communication strategies among service providers and the general public. Barrier: negative external attitudes and structures	Responsive, flexible, and long-term support for PWA needed for personal recovery and living successfully with aphasia and providing opportunities to participate autonomously.
Norman 2023 (43)	ScopR n/r	ABI (incl stroke) PLWBI, carer/family, HP (n/r)	Fatigue, pain, emotional, behavioral, cognition, physical, RTW or school or avocational, support, housing adaptations, education, and information	Living, leisure, health	Facilitators: specific goals, information at all stages, active involvement in decision making	Need multiple long-term services in community, comprehensive range, well-trained HPs. Needs service coordination
Ramsberg 2023 (44)	SR 7	Stroke (in last 2 years of life) PLWS (2,175)	Assessment and management of end-of-life symptoms; e.g., dysphagia and dysarthria plus non-stroke issues of pain, dyspnoea, constipation, and psychological needs	Participation	Facilitators: management of pain established Barriers: limited access to specialists for dyspnoea, constipation and anxiety	Even though palliative and EoL guidelines exist, they are not being incorporated into care
Sedzro 2023 (45)	ScopR	Stroke Caregivers (n/r)	Physical, emotional, psychological and financial demands of caregiving	Reduced social life	None stated	Need to acknowledge caregivers’ values as well as their health and wellbeing needs
See Toh 2022 (46)	SR 19 (qual)	Stroke PLWS, carers (432)	Performing caregiving tasks; alterations to lifestyle and health; coping with caregiving, family roles and relationships; availability of informal support for caregivers; financial and healthcare resources; care coordination	Physical, psychological	Facilitator: health care professionals preparing caregivers, caregiver support Barriers: inadequate preparation, education and training, social isolation, not feeling prepared.	Lack of resources and coping impacts on caregiving roles during transitional care. Impacts person and family life. Role changes impact on wellbeing.
Shaik 2024 (16)	UmbSR 17	Stroke PLWS (5,623)	Unmet needs for unfamiliar body; enduring morbidity, disruptions to daily living, fragmented relationships, withering self-image	Self-esteem, relationships & engagement.	Facilitators: interpersonal relationships, intrapersonal strengths; compassionate health system; holistic rehab	PReTS model – **P**sycho-social **Re**habilitation **T**rajectory of **S**troke survivor.
Sorbello 2023 (47)	ScopR	Stroke (vision impairment) PLWS (n/r)	Physical, vision impairments—unmet needs in care provision, particularly information, referral and long-term management	Vision (health)	Facilitators: Information, referrals, long-term management plan. Barriers: hidden disability, lack of awareness or support	Post-stroke vision impairment pathways are varied and not standardized.
Tellier 2009 (22)	SR 13	Stroke (mild) PLWS (226)	Lack of access to rehab services by mild stroke survivors	Physical health is impacted—deconditioning and difficulty in reengagement back to the community	Barrier: sent home with no referral to rehab services	The impact of mild stroke on family’s needs to be explored
Temehy 2022 (48)	mixedR 27	Stroke PLWS and Carers (n/r)	Limited suitable information; inadequacy of care and services	Psychological, physical, social, rehabilitation, and other needs	None stated	Literature dominated by Western data—need more from Asian and Middle Eastern
Torre 2022 (17)	UmbSR	Stroke (of working age) PLWS (n/r)	Physical, emotional, social	Return to work	Facilitators: external support, independent in ADLs, better cognition, previous role was non-manual Barriers: fatigue, psychiatric, cognitive, or physical disability, inadequate services, previous manual	Many important factors impact RTW—should address barriers and facilitators
Wray 2017 (19)	SR 32	Stroke (communication difficulties) PLWS (518)	Communication needs; meaningful role; maintaining social support	Social isolation, loss of friendships, self-identity, avoiding social interactions, confidence, feeling valued; impact on QoL, loss of purpose, loss of previous activities, emotional distress—sense of loss, grief.	Facilitator: self-management interventions appropriate Barrier: lack of network support for this group of patients	None relevant
Zawawi 2020 (49)	SR 105	Stroke PLWS and Carers (n/r)	Physical & other stroke-related problems (physical; cognitive and emotional functions); social participation (support in living; community re-integration; relationships); information (stroke related; post stroke care and rehab; being productive and continue living after stroke); rehabilitation and care (rehab; health-related care)	Health, social engagement, and meaning, relationships	None stated	None relevant
Zhang 2022 (21)	SR 12	Stroke (young) PLWS (178)	Unmet rehab needs (age-adapted) including information; social reintegration, returning to work	QoL; social isolation, self-confidence; value; emotions (shame, fear); social reintegration problems	Facilitator: adapting and developing coping skills to participate socially	None relevant

N, number of included studies; ScopR, scoping review; PLWS, person living with stroke, n/r, not reported; HPs, health professionals; Qual, qualitative; SR- systematic review; PLWBI, person living with brain injury; NZ, New Zealand; UmbSR, umbrella systematic review; RTW, return to work; LR, literature review; QoL, quality of life; Incl, including; RER rapid evidence review; MDT, multidisciplinary team; SLP, speech and language therapist; OT, occupational therapist; ABI, acquired brain injury; EoL, end of life; rehab, rehabilitation; mixedR, mixed methods review; ADLs, activities of daily living.

Twelve reviews examined the perspective of the person with the stroke, four focused on the perspective of caregivers, and 12 were mixed. Authors of most studies investigated the population of people with stroke (25 reviews), and 3 were mixed populations of stroke and traumatic brain injury. Considering demographic sub-groups, three focused on specific regional populations (Africa and New Zealand, south-east Asian diaspora), one focused on unmet needs among younger people (<45 years), one on people with mild stroke, and one on people with limited English. Condition-related subgroups comprised three reviews focussing on people with post-stroke aphasia/communication issues, and one for people with visual impairments. In addition, two focussed specifically on people post-stroke returning to work and one on people who were in the last 2 years of life after stroke.

In 10 reviews, the authors did not state the time since stroke; of those who did, 12 included participants in the chronic stage (3 or more months after diagnosis) and six in the sub-acute (4–12 weeks after diagnosis). No reviews focussed exclusively on transition times.

### Overlap of included reviews

The overlap analysis comprised 25 reviews of primary studies, spanning 648 unique primary studies and 752 inclusions, yielding a CCA of 0.67% (slight overlap; 0–5% band, Pieper et al. (13)).

Three further eligible reviews were overviews of reviews and are summarized separately. Choo et al. (15) synthesized 10 systematic reviews (referencing 187 primary studies) on psycho-socio-emotional challenges in stroke caregiving; Shaik et al. (16) synthesized 17 reviews (400 primary studies) on stroke survivors’ psychosocial recovery needs; and La Torre et al. (17) synthesized 24 reviews on return to work after stroke. At the level of their included reviews, the three overviews overlapped only modestly with one another: five reviews each appeared in two overviews, and none appeared in all three. Two reviews included within the overviews—Manning et al. (18) and Wray & Clarke (19)—were themselves included reviews in the present synthesis, illustrating the nested relationship between the overviews and the primary-study reviews.

### Overview of unmet needs

The extracted unmet needs were summarized into 57 common items, which were further ascribed to the seven categories of pathology (health condition), impairment (physical or psychological), activity limitation, participation restriction, or environmental factors (differentiating general environment from services), as defined in the ICF (10). If individual needs were noted more than once, they were listed again to ensure that we had a relative frequency of the largest of the unmet needs. Table 2 shows the individual studies and the presence of unmet needs by ascribed ICF categories.

**Table 2:** Included reviews with reported unmet needs as ascribed to the ICF-WHO categories and reported impact on wellbeing indicators.

Study/review first author [ref]	Subgroup	Pathology (B)	Physical (B)	Psychological (P)	Activity (BPS)	Participation (BPS)	Environment	Services	Competence (BPS)	emotional stability (P)	Engagement (S)	Meaning (P–S)	Optimism (P)	positive emotions (P)	Relationships (S)	Resilience (P)	Self-esteem (P)	Vitality (B–P)
		Unmet needs - ICF Framework						Negative impact on wellbeing 10-item indicators										
Assefa (32)	Africa																	
Bright (33)	NZ																	
Chen (6)	General																	
Choo (15)	Carers																	
Clark (20)	Limited English																	
Connor (34)	General																	
Coutts (35)	Working age																	
Denham (36)	Carers																	
Greenwood (37)	Carers																	
Guo (14)	General																	
Halabi (38)	General																	
Kokorelias (39)	SE Asian																	
Kristo (40)	Aphasia																	
*Lafiatoglou* (41)	General																	
*Laurie* (42)	After discharge																	
Manning (18)	Aphasia																	
*Norman* (43)	General																	
Ramsberg (44)	EoL																	
Sedzro (45)	Carers																	
See Toh (46)	Carers																	
Shaik (16)	General																	
Sorbello (47)	Vision																	
Tellier (22)	Mild stroke																	
Temehy (48)	General																	
Torre (17)	Working age																	
Wray (19)	Communication																	
Zawawi (49)	General																	
Zhang (21)	Working age																	
	Frequency	7	12	13	7	12	5	16	3	10	19	14	1	7	17	1	9	6

(B) – Biological component of the Biopsychosocial (BPS) model; (P) psychological component of BPS; (S) – social component of BPS; ICF: WHO – World Health Organisation International Classification of Functioning; NZ – New Zealand; general—included general stroke population with no sub-group focus; EoL, end of life; SE, South East.

Empty cells indicate no reporting of unmet needs in these ICF domains or wellbeing indicators.

To enable comparison of needs and wellbeing across groups—general stroke population (no subgroups specified) is shaded in dark grey; subgroups related to geography, culture, or language are in yellow; people with aphasia in green; carers in blue; working age in light grey; miscellaneous subgroups in orange.

Italics: “acquired brain injury” population—combined stroke/traumatic brain injury.

As shown in Table 2, the largest number of reported unmet needs fit within the Services aspect of ‘Environment’ and included lack of information, access to relevant and appropriate rehabilitation, training, and education. The second most frequent was in the ‘psychological impairments’ part of the framework and included unmet needs for mental health, adjustment, cognitive, and behavioral changes. Specific stroke care was the largest unmet need in pathology; general unmet needs for physical impairments; dependence on ADLs in activity; quality of life, life roles (such as return to work and social roles), and community engagement in the participation category; and support for a healthy lifestyle in the environment. The included frequency counts (Table 2) are only an indication of reporting, not of certainty or strength of evidence.

### Impact on health and wellbeing

All included articles provided sufficient information to extract data on the impact of unmet needs on the health and wellbeing of the population. These were mapped onto the 10-item multi-dimensional indicators of wellbeing described by Ruggeri et al. (11) In some instances, the wellbeing indicators had been reported as ‘unmet needs’. Most reported that the biggest impact was on ‘engagement,’ which encompasses participation in life roles and activities, followed by ‘meaning’ or satisfaction found in that engagement. These two indicators are often paired to describe ‘quality of life’. In many reviews, the authors also identified significant negative impacts on relationships (having people who care about you and/or who support and help you), followed by impacts on emotional and mental health (such as reduced emotional stability or positive emotions), as well as a reduced sense of self (self-esteem and identity), along with shame and embarrassment, and a reduced sense of meaning or value that led to decreased social ability (relationships) and confidence. Financial impacts also affected their ability to engage in activities. Many authors also described specific impacts on physical wellbeing (vitality), such as disturbed sleep quality and efficiency. Figure 2 also depicts the extracted items, which were themed and mapped into a broad biopsychosocial framework.

**Figure 2:**
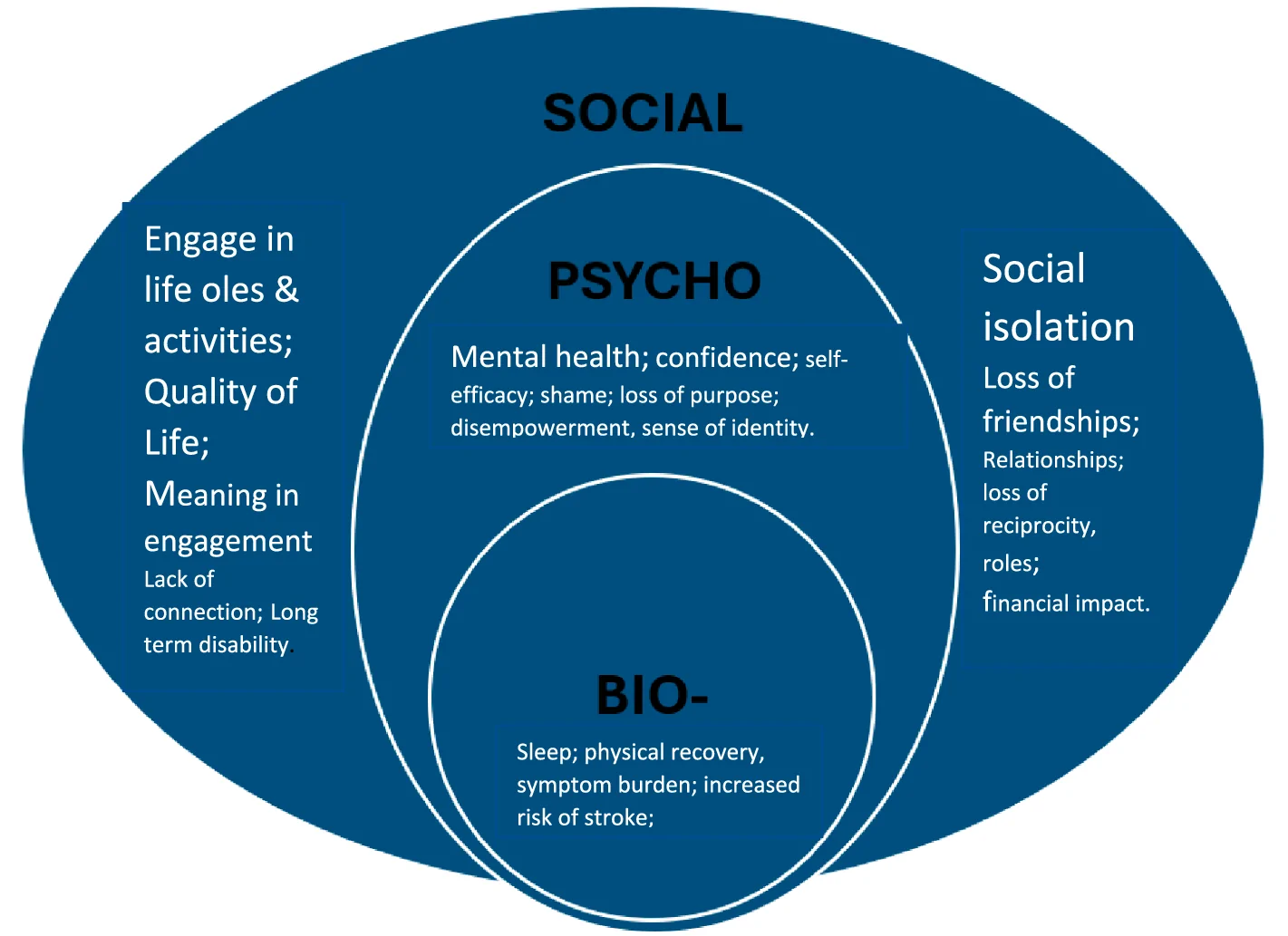
Mapping of impact of unmet needs on health and wellbeing using a biopsychosocial model. Larger type indicates more frequent reporting.

### Barriers and facilitators to reduce unmet needs or their impact

Facilitators were sometimes expressed as personal attributes like coping styles, the ability to recalibrate and adapt, and find meaning, and as proximal attributes like having supportive family and friends. (see Table 1). Many of the reviews made strong conclusions about facilitators from a service perspective; for example, where the unmet need related to the lack of provision of a service, then clearly those named services or interventions needed to be provided. These included services such as mental health support, respite, assessment/identification, provision of equipment and accessible environments, transport options, community resources, referral services, and more specific interventions and comprehensive rehabilitation programs. Authors of several reviews focussed on the need for education, knowledge, information and training as facilitators. Review authors noted that specific, dedicated assessments carried out regularly to identify unmet needs (and their impact) would be beneficial. Highly trained and experienced health professionals were often cited as facilitators.

There was also a strong theme relating to the need to support people with stroke to facilitate adjustment to their new circumstances, develop confidence and autonomy, and foster coping skills in the long term. This theme also extended to a call to increase the knowledge, awareness, and acceptance of family and society more broadly as facilitators of improved health and wellbeing. It was also noted that facilitating networks and peer support would reduce the impact of unmet needs, while the evidence for specific liaison workers and care coordinators was mixed.

Barriers to meeting the needs of people after stroke include lack of recognition of the specific needs of sub-groups such as culturally and linguistically diverse (CALD) communities (e.g., with lack of interpreters), younger stroke survivors (different goals and needs – especially those related to returning to work), and those with communication issues. Several reviews highlighted the significant barriers in the community around stigma and negative attitudes towards brain damage, as well as the potentially negative impact of some faith and cultural groups.

### Predictive modeling and multi-modal approaches

None of the included review articles had predictive modeling or multi-modal approaches to exploring various unmet needs and the impact on health and wellbeing.

## Discussion

This scoping review of reviews has provided new insights into the breadth of unmet needs for people after stroke. Importantly, we synthesized the evidence base to better understand how these unmet needs affect the health and wellbeing of these populations, and confirmed that these needs vary across sub-groups. We have identified gaps in the literature and in our understanding of unmet needs to be able to make more targeted and comprehensive recommendations for future research and future care.

The authors of the review articles reported an overall paucity of high-quality studies, and a lack of clarity around the impact of absent or reduced long-term therapy or services. While many studies have investigated unmet needs, few have linked these needs to cost-effective interventions that may reduce their impact. There was also a reported lack of studies into specific areas such as sleep, minority groups (CALD and young stroke), looking at the ‘true’ quality of life of both the person with stroke and their caregivers, and considering environmental factors in the context of what the survivors valued in terms of activities. There was a strong focus on psychosocial aspects that would increase people’s acceptance, autonomy, and agency, and that would counter the illusory and unfulfilled goals of ‘returning to normal’. Age-adapted support for social reintegration and an acknowledgment of the hidden or invisible stroke symptoms were also highlighted.

### Specific findings

Unmet needs after stroke were generally well characterized, particularly for the sub-group with communication issues, focusing on unmet needs for understanding, facilitating relationships, and environmental issues such as lack of external support, acceptance, and understanding of impaired communication. With a broad representation from across the globe, there is clearly a lot of evidence that unmet needs after stroke span the spectrum from specific stroke care to impairments of body function/structure (physical and psychological), restrictions on activities of daily life (dependence) and limitations in participation in life (reduced social integration and meaningful roles). Unsurprisingly, the review focussing on people with limited English proficiency (in English-speaking countries) (20) found that unmet needs were compounded by a lack of interpreters to explain stroke issues, services and recovery. Similarly, the reviews focussing on young stroke survivors (17, 21) highlighted specific high needs for vocational and marital support in their recovery journey. People who had a mild stroke were reported to generally miss out on services (22).

The most often reported sub-group—caregivers of people with stroke—consistently were reported as having high unmet needs related to the burden, skills in caring, and the resulting impact on their social, emotional, and physical wellbeing. A previous study confirmed the interdependence between people with stroke and their caregivers, reporting that the odds of a caregiver experiencing negative impacts increase with the number of unmet needs of the stroke survivor (23).

### Impact on health and wellbeing

We were particularly interested in exploring how unmet needs are reported and their perceived impact on health and wellbeing. For a considerable time, the Preamble to the WHO Constitution has defined health as a “state of complete physical, mental and social wellbeing and not merely the absence of disease or infirmity” (WHO) (24, 25). Furthermore, the WHO has identified the determinants of health as including the social and economic environment, the physical environment and the person’s individual characteristics and behaviors (26). While their definition of health encompasses wellbeing, others have described the two concepts as different yet closely related. For example, Steptoe et al. (27) explain that although the two concepts have a bidirectional relationship, subjective wellbeing is affected by many factors other than health. These include material conditions, social and family relationships, and social roles and activities. The key point is that, despite a long history of understanding health and wellbeing in this holistic way, our review confirms that unmet needs following stroke still clearly affect these factors.

Furthermore, more subtle definitions of wellbeing are also reflected in our findings—for example, the three categories of subjective wellbeing included evaluative (satisfied with life), hedonic (feelings of happiness, versus sadness or stress or pain), or eudaimonic (sense of purpose and meaning in life) (27). Of these, the sense of engagement and meaning was highlighted most strongly as loss of engagement, loss of roles, and loss of self-identity.

Our findings also require an unpacking of the frequent reporting of the impact of unmet needs on ‘quality of life’ (QoL). Quality of life can be considered to have both subjective judgments and objective factors. Various definitions of QoL cover human needs, expectations, preferences, satisfaction, flourishing and hedonism, alongside physical, material, social and emotional wellbeing and “the extent of personal development and purposeful activity, all weighted by a personal set of values” (28). We use the term as reported by the authors of the included articles as a concept seemingly separate from other specific reports of impact (for example on reduced relationships or loss of engagement and meaning). However, from the accepted and broad-ranging definitions of QoL, it could be argued that all unmet needs and their impact load onto this one term. An association between increased unmet needs and reduced QoL has been previously reported in chronic stroke (29).

A third lens on QoL considers only aspects that are affected by health—“those aspects of self-perceived wellbeing that are related to, or affected by, the presence or absence of disease” (30). The field of health economics has added a fourth dimension to Health Related (HR) QoL by ascribing a value (usually economic) to different health states (31). Karimi and Brazier report on the inappropriate conflation of the terms ‘health’, ‘health-related quality of life’ and ‘quality of life’—meaning they are inappropriately used interchangeably rather than seen as interdependencies (31). Given the WHO definition of health, which includes the bio-psycho-social framework, *health-related* QoL can be considered how well someone functions in their physical, mental, and social life domains. These authors specify that non-health factors should not be included in HR-QoL, for example, economic or political circumstances.

### Recommendations

From our synthesis of reported unmet needs and their impact on health and wellbeing after stroke, we recommend using the best available evidence to address gaps in our understanding, guide future research, and plan and deliver future care.

First, we would recommend that future studies limit charting unmet needs and their impact on the population of people with stroke unless they also address actionable outcomes—these have been sufficiently noted with a saturation of findings. The addition of 12 reviews since 2022 suggests a measure of research waste. Rather, future studies should examine the effectiveness and cost-effectiveness of addressing these unmet needs. In particular, the frameworks used in this review (the WHO ICF and the biopsychosocial model more broadly) highlight that any interventions to address these gaps need to be system-based—that is, work with the individual in the context of their daily life and in their environment (community and health service). However, we may need to develop a specific framework to cover all facets of wellbeing impacted by stroke, particularly those emerging in the literature. Facilitators and barriers to these needs and impacts often reside in individual and community understanding, and across physical, psychological, and social realms. Ultimately, interventions should align with broader social concerns of integration, life meaning, and sense of self.

Subgroups such as those with communication issues, young onset, or from CALD groups need special attention to ensure they have equitable access to relevant services, as well as support and understanding from the community.

Finally, better tools and frameworks to accurately identify health and wellbeing issues after stroke, and the cost implications of attending (or not attending) to unmet needs contributing to these issues, are urgently required.

### Strengths and limitations

The strength of this review of reviews is in the breadth of international evidence found across all groups of people with stroke. The scoping nature of the review ensured we achieved the greatest possible understanding of the current evidence base, and could identify both unmet needs and impacts that apply generally across the stroke population, as well as those that might be specific to sub-groups. Due to the early commencement of this review, we did not include non-English reviews as ready translation was not then feasible. Future reviews can now make use of more accessible translation services.

The considerable heterogeneity of the individual studies within the reviews is both a strength and a limitation—applying our findings to local contexts, cultural factors, and health systems and policies will require further consideration.

A limitation is that we did not evaluate the quality of individual reviews; however, establishing the scope of work reported to date was the primary aim. Instead, we included the level of evidence by review type to enable the reader to consider the strength of the evidence. The included reviews reported poor quality in many of their primary studies. Therefore, future studies that attempt to further our understanding of these groups should focus on sampling, recruitment, and breadth of investigation, particularly in emerging areas such as shame and self-efficacy. Current low frequency of reporting does not mean low importance.

Furthermore, three eligible reviews were overviews of reviews and could not be incorporated into the primary-study CCA without mixing units of analysis; their exclusion means the reported overlap reflects reviews of primary studies only, and any overlap occurring at the level of secondary syntheses is described narratively rather than quantified.

A final limitation is the considerable conceptual overlap between the classifications of unmet needs, impact on life, and wellbeing. We report the original data extracted from the reviews for readers to assess the veracity of our synthesis.

## Conclusion

We have identified consistent unmet needs reported across the international stroke populations and confirm that these span the spectrum from body structure and function through to independent activities and life roles. We have furthermore confirmed that these unmet needs are not trivial and impact major domains of wellbeing across the physical, psychological and social framework. Strong evidence that interventions and services must address the breadth of needs (and their impact), adapted for sub-groups, and must be comprehensive and appropriately evaluated. Future studies should advance the field with solutions rather than continue reporting data on unmet needs.

## Data Availability

Publicly available datasets were analyzed in this study. The data can be found in the reference list.

## Appendix 1: Example of Search strings

1. Brain Injuries, Traumatic/ or Brain Injuries/ or Brain Damage, Chronic/ or Ischemic Stroke/ or Stroke, Lacunar/ or Ischemic Stroke/ or Embolic Stroke/ or Hemorrhagic Stroke/ or Thrombotic Stroke/ or Stroke/ or Brain Infarction/ or Brain Ischemia/ or Brain Stem Neoplasms/ or Hypoxia-Ischemia, Brain/ or Brain Hemorrhage, Traumatic/ or Brain Stem Infarctions/ or Brain Injury, Chronic/ or Hypoxia, Brain/ or Brain Stem Hemorrhage, Traumatic/ or Brain Neoplasms/
2. (“brain tumour” or “brain cancer” or “acquired brain injury” or stroke or “post stroke” or “post-stroke” or poststroke or “cerebrovascular accident” or CVA or “traumatic brain injury” or TBI or “brain injury”) (ti,ab,kf)
3. 1 OR 2
4. (“community dwelling person*” or “community living” or Community or home or “community dwelling” or “independent living” or outpatient* or independent) (ti,ab,kf)
5. Independent Living/ or Outpatients/
6. 4 OR 5
7. (“health care need” or “health care needs” or “supportive care need” or “supportive care needs” or “medical care need” or “unmet medical need” or need or needs or (unmet adj2 need) or demand* or requirements* or facilitator* or barrier*) (ti,ab,kf) or “Health Services Needs and Demand”/
8. “Quality of Life”/ or Health/
9. (“quality of life” or QOL or wellbeing or wellbeing or “health impact*” or “health outcome*”) (ti,ab,kf)
10. 8 OR 9
11. (“systematic review” or review or meta-analysis or “meta analysis”) (ti,ab,kf)
12. “Systematic Review”/ or “Review”/ or Meta-Analysis/
13. 11 OR 12
14. 3 AND 6 AND 7 AND 10 AND 13
15. limit 14 to (english language and yr = “2000 -Current”).
